# Estimating and mapping ecological processes influencing microbial community assembly

**DOI:** 10.3389/fmicb.2015.00370

**Published:** 2015-05-01

**Authors:** James C. Stegen, Xueju Lin, Jim K. Fredrickson, Allan E. Konopka

**Affiliations:** Fundamental and Computational Sciences Directorate, Biological Sciences Division, Pacific Northwest National LaboratoryRichland, WA, USA

**Keywords:** ecological niche theory, ecological neutral theory, Hanford Site 300 Area, microbial biogeography, null modeling, phylogenetic beta-diversity, phylogenetic signal, Raup–Crick

## Abstract

Ecological community assembly is governed by a combination of (i) selection resulting from among-taxa differences in performance; (ii) dispersal resulting from organismal movement; and (iii) ecological drift resulting from stochastic changes in population sizes. The relative importance and nature of these processes can vary across environments. Selection can be homogeneous or variable, and while dispersal is a rate, we conceptualize extreme dispersal rates as two categories; dispersal limitation results from limited exchange of organisms among communities, and homogenizing dispersal results from high levels of organism exchange. To estimate the influence and spatial variation of each process we extend a recently developed statistical framework, use a simulation model to evaluate the accuracy of the extended framework, and use the framework to examine subsurface microbial communities over two geologic formations. For each subsurface community we estimate the degree to which it is influenced by homogeneous selection, variable selection, dispersal limitation, and homogenizing dispersal. Our analyses revealed that the relative influences of these ecological processes vary substantially across communities even within a geologic formation. We further identify environmental and spatial features associated with each ecological process, which allowed mapping of spatial variation in ecological-process-influences. The resulting maps provide a new lens through which ecological systems can be understood; in the subsurface system investigated here they revealed that the influence of variable selection was associated with the rate at which redox conditions change with subsurface depth.

## Introduction

In his conceptual synthesis, Vellend (2010) proposed that ecological communities are influenced by a combination of ecological selection, organismal dispersal, ecological drift, and speciation (see also Hanson et al., 2012). This is a useful perspective that places all ecological communities within the same conceptual framework, thereby facilitating cross-system comparisons. While there is some knowledge of what causes shifts in the relative influences of governing processes (e.g., Horner-Devine and Bohannan, 2006; Chase, 2007, 2010; Stegen et al., 2012), turning Vellend’s conceptual framework into an operational framework is a significant challenge. As an initial attempt, Stegen et al. (2013) proposed a null modeling approach that estimates the relative influences of ecological components within Vellend’s framework at the scale of a metacommunity. To provide context, we next introduce the concepts synthesized in Vellend (2010).

**FIGURE 1:**
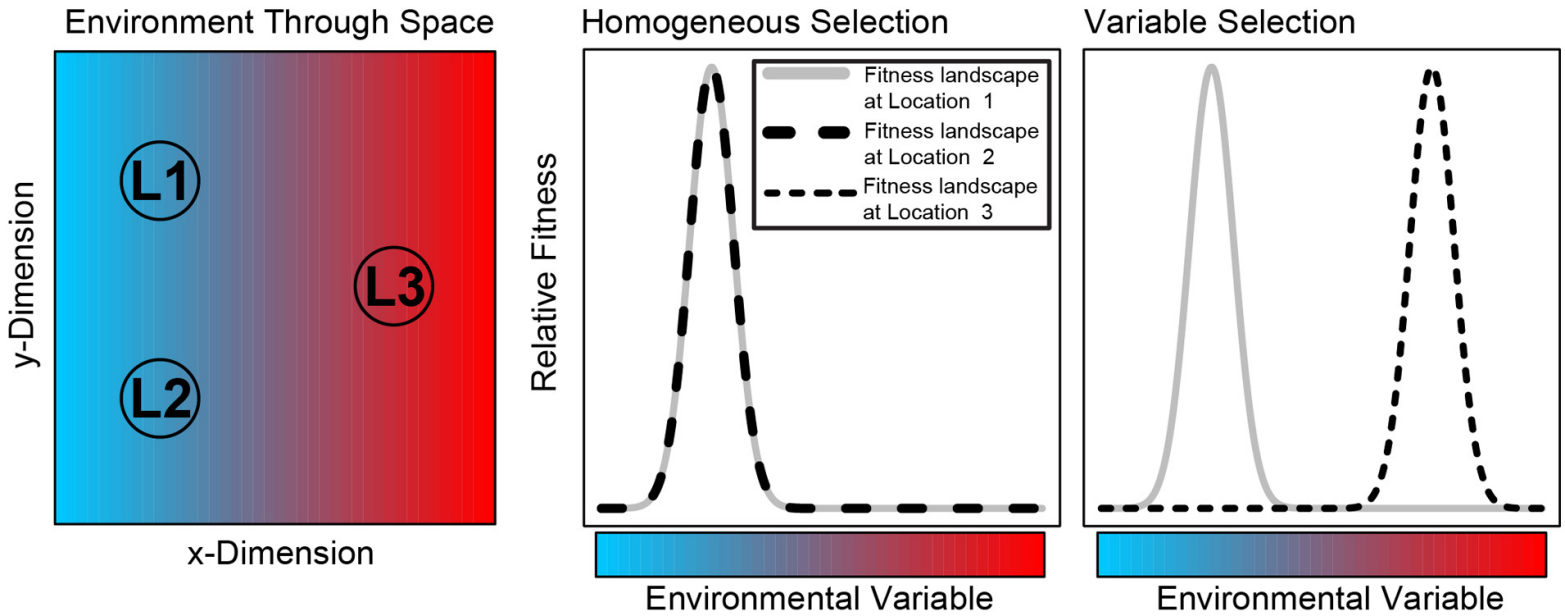
**Conceptual summary of homogeneous selection and variable selection.** The **left panel** shows spatial variation in an arbitrary environmental variable. For illustration, we might consider temperature to be the environmental variable, going from cold (blue) to hot (red). There are three locations indicated as L1, L2, and L3; L1 and L2 are spatially separated, but they are both in a cold environment; L3 is in a hot environment. The **center panel** shows the fitness landscapes at L1 and L2; they are identical because the environment is the same; species adapted to cold temperatures will have higher fitness than species adapted to hot temperatures. In turn, the selective environment is consistent—between L1 and L2—such that we expect cold-adapted species to comprise communities found in both locations. This is an example of ‘homogeneous selection.’ The **right panel** shows fitness landscapes in L1 and L3; because of different temperatures, the fitness landscapes do not overlap each other; cold adapted species are expected in L1 and hot-adapted species are expected in L3. In this case, the selective environment varies through space; this is an example of ‘variable selection.’

Ecological selection results from different organisms having different levels of fitness for a given set of environmental conditions; here we consider ‘environmental conditions’ to include both abiotic variables (e.g., temperature) and biotic factors related to organismal interactions. If environmental conditions are homogeneous through space, the selective environment will also be homogeneous (Vellend, 2010). The scenario in which a consistent selective pressure—that results from consistent environment conditions—is the primary cause of low compositional turnover is referred to as ‘homogeneous selection’ (**Figure 1**), as in Dini-Andreote et al. (2015). On the other hand, if environmental conditions change through space, among-taxa fitness differences can change (Vellend, 2010). The scenario in which a shift in selective pressure—that results from a shift in environment conditions—is the primary cause of high compositional turnover is referred to as ‘variable selection’ (**Figure 1**).

Organismal dispersal refers to the movement of organisms through space (Vellend, 2010). High dispersal rates have the potential to homogenize community composition such that there is low turnover in composition (Leibold et al., 2004). High dispersal rates can, in principle, overwhelm selection-based influences, a scenario at the population level known as source-sink dynamics (Dias, 1996). The scenario in which a high dispersal rate—between a given pair of communities—is the primary cause of low compositional turnover is referred to as ‘homogenizing dispersal’ (Stegen et al., 2013).

On the other hand, when selection is relatively weak and organisms rarely move between communities, ecological drift (i.e., stochastic changes in population sizes) can lead to marked differences in community composition (Hubbell, 2001; Vellend, 2010). The scenario in which high turnover in composition is primarily due to a low rate of dispersal enabling community composition to drift apart is referred to here as ‘dispersal limitation.’ To be clear, our use of the term ‘dispersal limitation’ does not directly refer to a low rate of dispersal between a given pair of communities. We use ‘dispersal limitation’ to indicate a situation in which a low dispersal rate is the *primary cause* of high compositional turnover; differences in selective environments may be the primary cause of high compositional turnover even when dispersal rates are low, which would fall within the conceptualization of ‘variable selection.’

It is important to consider that the rate of dispersal and strength of selection are continuous variables with context-dependent magnitudes and that these processes simultaneously influence ecological communities. The above-summarized scenarios assume relatively extreme levels of dispersal and/or selection, but it is likely that some natural systems (or parts of systems) are characterized by moderate levels of dispersal and selection. In this case, neither process may dominate, leading to compositional differences between communities that are due to a mixture of stochastic organismal movements and stochastic birth–death events. Stegen et al. (2013) used the term ‘ecological drift’ to refer to this scenario, but here we use the term ‘undominated’ to refer more directly to the scenario in which neither dispersal nor selection is the primary cause of between-community compositional differences.

Here we first develop and test an extended version of the Stegen et al. (2013) framework and, in turn, apply the extended framework to subsurface microbial communities distributed across two geologic formations within the 300 Area of the U.S. Department of Energy’s Hanford Site in southcentral Washington State. Our framework uses null models to estimate the degree to which compositional turnover between a single focal community and all other sampled communities is governed by different ecological processes (homogeneous selection, variable selection, homogenizing dispersal, and dispersal limitation). The framework also estimates the portion of compositional turnover that is not dominated by a single process. To evaluate this framework we simulated community assembly under different scenarios; the analytical framework was applied to simulated communities to ask how robust the framework was in detecting the dominance of specific ecological processes. In turn, process estimates were generated for the field-sampled subsurface communities; these estimates were combined with environmental data and community spatial locations to identify environmental drivers and to characterize spatial variation in the relative influence of each ecological process.

## Materials and Methods

### Subsurface Sampling

The dataset used here is the same as in Stegen et al. (2013) such that here we provide a brief summary of methods used to generate those data. We analyzed sediment-associated bacterial communities found within two subsurface geologic formations roughly 250 m from the Columbia River in Richland, WA, USA. The sampled communities were within the Hanford Integrated Field Research Challenge (IFRC) site^1^, and were sampled during the drilling of 26 IFRC wells (Bjornstad et al., 2009); supplemental material in Stegen et al. (2013) provides details on sample locations. The sampled formations are structurally and hydrologically distinct, are separated vertically, but are in the same horizontal location; 16 communities were sampled within the deeper-lying Ringold Formation, which has restricted water flow and finer-grain sediments; 28 communities were sampled within the shallower Hanford formation, which has less restricted water flow and coarser-grain sediments (Bjornstad et al., 2009). In addition, the Ringold formation changes with depth from oxidized to reduced conditions (Bjornstad et al., 2009). Microbial communities were characterized through pyrosequencing of the 16S rRNA gene (see Lin et al., 2012a,b), DNA sequences were processed using QIIME (Caporaso et al., 2010), and statistical analyses were done using R^2^ (for molecular and bioinformatics methods see Stegen et al., 2013). Each microbial community was associated with environmental metadata including its spatial position, its horizontal distance from the Columbia River, the elevations of reduced and oxidized portions of the Ringold Formation, and the vertical thickness of the oxidized portion of the Ringold. Vertically structured features were obtained from Bjornstad et al. (2009).

### Statistical Approach

Our statistical framework uses phylogenetic turnover between communities to infer ecological selection (Stegen et al., 2013). This approach requires significant ‘phylogenetic signal’ (Losos, 2008) whereby there is a relationship between phylogenetic relatedness and ecological similarity (Kraft et al., 2007; Cavender-Bares et al., 2009; Fine and Kembel, 2011); as in other microbial systems (e.g., Wang et al., 2013) this was found to be true such that more closely related taxa were more similar ecologically (see Figure 2 in Stegen et al., 2013).

To quantify phylogenetic turnover between communities we used the between-community mean-nearest-taxon-distance (βMNTD) metric (Fine and Kembel, 2011; Webb et al., 2011), which quantifies the phylogenetic distance between each species in one community (*k*) and its closest relative in a second community (*m*):

$$\beta MNTD=0.5[\Sigma_{ik=1}^{n_{k}}f_{ik}\,\;\,\;\min\,\;\;\left(\Delta_{i_{k}j_{m}}\right)+\Sigma_{i_{m}=1}^{n_{m}}{fi}_{m}\,\,\;\min\,\;\left(\Delta i_{m}j_{k}\right)],$$

where f_ik_ is the relative abundance of species *i* in community *k*, *n_k_* is the number of species in *k*, and min (Δ_i_k_j_m__) is the minimum phylogenetic distance between species *i* in community *k* and all species *j* in community *m*. βMNTD was calculated using the R function ‘comdistnt’ (abundance.weighted = TRUE; package ‘picante’).

For each pair of communities within each geologic formation we generated the value of βMNTD expected if ecological selection was *not* the primary factor governing compositional differences by randomly shuﬄing species across the tips of the phylogeny and, recalculating βMNTD. This procedure was repeated 999 times to provide a distribution of null values. If the observed βMNTD value was significantly (a) less or (b) greater than the null expectation, we inferred that (a) homogeneous selection or (b) variable selection was responsible for the similarity or differences, respectively, between the pair of communities. Significance was evaluated via the β-Nearest Taxon Index (βNTI), which expresses the difference between observed βMNTD and the mean of the null distribution in units of SDs; βNTI values < -2 or > +2 indicate significance (Stegen et al., 2012). These interpretations of the βNTI metric were evaluated and supported via simulation modeling, as described below.

If observed βMNTD does not significantly deviate from the null expectation it indicates that the observed compositional difference is not due to selection (Hardy, 2008), and should therefore be due to very low rates of dispersal (i.e., dispersal limitation), very high rates of dispersal (i.e., homogenizing dispersal), or a lack of dominance between selection and dispersal (i.e., the ‘undominated’ scenario described in the Introduction). To distinguish among these possibilities, we used the Raup–Crick metric (Chase et al., 2011) extended to incorporate species’ relative abundances (as in Stegen et al., 2013); referred to as RC_bray_. Like βNTI, RC_bray_ was based on a comparison between observed and expected levels of turnover, but without using phylogenetic information.

To generate an expected degree of turnover—for use in quantifying RC_bray_—we drew species into each local community until empirically observed local species richness was reached. The probability of drawing a given species was proportional to the number of local sites occupied by that species (as in Chase et al., 2011). In turn, we drew individuals into each selected species until total abundance—summed across species—was equal to observed total abundance. The probability of drawing an individual into a given species was proportional to the relative abundance of that species across all local communities (as in Stegen et al., 2013). This procedure represented stochastic assembly of local communities under the assumptions of weak selection and random dispersal. Compositional differences were quantified with Bray–Curtis (Bray and Curtis, 1957). Repeating stochastic assembly 999 times for each pair of communities provided a null distribution of Bray–Curtis dissimilarities.

To compare observed Bray–Curtis to the null distribution we followed Chase et al. (2011). The number of comparisons between randomly assembled communities that had a Bray–Curtis value greater than the empirical Bray–Curtis was added to half the number of ties. This sum was standardized to range from -1 to +1 by subtracting 0.5 and multiplying by 2; the resulting value is RC_bray_; values between -0.95 and +0.95 indicate that compositional turnover between a given pair of communities is ‘undominated.’ In turn, we infer that RC_bray_ values <-0.95 or >+0.95 indicate that selection, dispersal limitation, or homogenizing dispersal govern observed compositional differences. Furthermore, dispersal limitation is expected to increase differences in community composition and should therefore result in RC_bray_ values greater than +0.95. Homogenizing dispersal, in contrast, increases compositional similarity and should therefore result in RC_bray_ values less than -0.95 (Stegen et al., 2013). These interpretations of the RC_bray_ metric were evaluated and supported via simulation modeling, as described below.

### Quantifying Influences of Ecological Processes

The composition of each community was compared to the composition of all other sampled communities within the same geologic formation. For a given community we estimated the relative influence of variable selection or homogeneous selection as the fraction of its comparisons with βNTI > +2 or βNTI < -2, respectively. Selection is excluded as the dominant process when |βNTI| < 2; in these cases RC_bray_ >+0.95 or < -0.95 were taken as evidence that dispersal limitation or homogenizing dispersal, respectively, was the dominant process. For a given local community the relative influence of dispersal limitation was therefore estimated as the fraction of its between-community comparisons with |βNTI| < 2 and RC_bray_ > +0.95. Similarly, the relative influence of homogenizing dispersal was estimated as the fraction of comparisons with |βNTI| < 2 and RC_bray_ < -0.95. The scenario where |βNTI| < 2 and |RC_bray_| < 0.95 indicates that neither selection nor dispersal strongly drive compositional turnover; this is the ‘undominated’ scenario and its relative contribution was estimated as the fraction of comparisons characterized by |βNTI| < 2 and |RC_bray_ | < 0.95. These interpretations of the βNTI and RC_bray_ metrics were evaluated and supported via simulation modeling, as described below.

### Statistical Models of Ecological-Process-Influences

In addition to estimating ecological-process-influences we aimed to map spatial variation in those influences. In the subsurface system studied here there were spatial locations where environmental features were characterized but where microbial communities were not. To predict ecological-process-influences across the entire system we first characterized explanatory variables using spatial and environmental data from all sampled locations.

The spatial positions of sampled locations were used with Principal Coordinates of Neighbor Matrices’ (PCNM, now referred to as ‘Moran’s Eigenvector Maps’) to describe spatial eigenvectors (function ‘pcnm’ in R package ‘vegan’; Borcard and Legendre, 2002; Borcard et al., 2011). The horizontal positions of locations at which microbial communities were sampled were unique to each formation such that spatial eigenvectors (referred to as PCNM axes) were generated independently for each formation. We also examined horizontally structured environmental features that included horizontal distance from the Columbia River, the elevations of the reduced and oxidized portions of the Ringold Formation, and the vertical thickness of the oxidized Ringold. Spatial eigenvectors and the four environmental features were used to construct statistical models for each ecological process in each formation; in all cases the statistical models were linear, multiple regression models.

**FIGURE 2:**
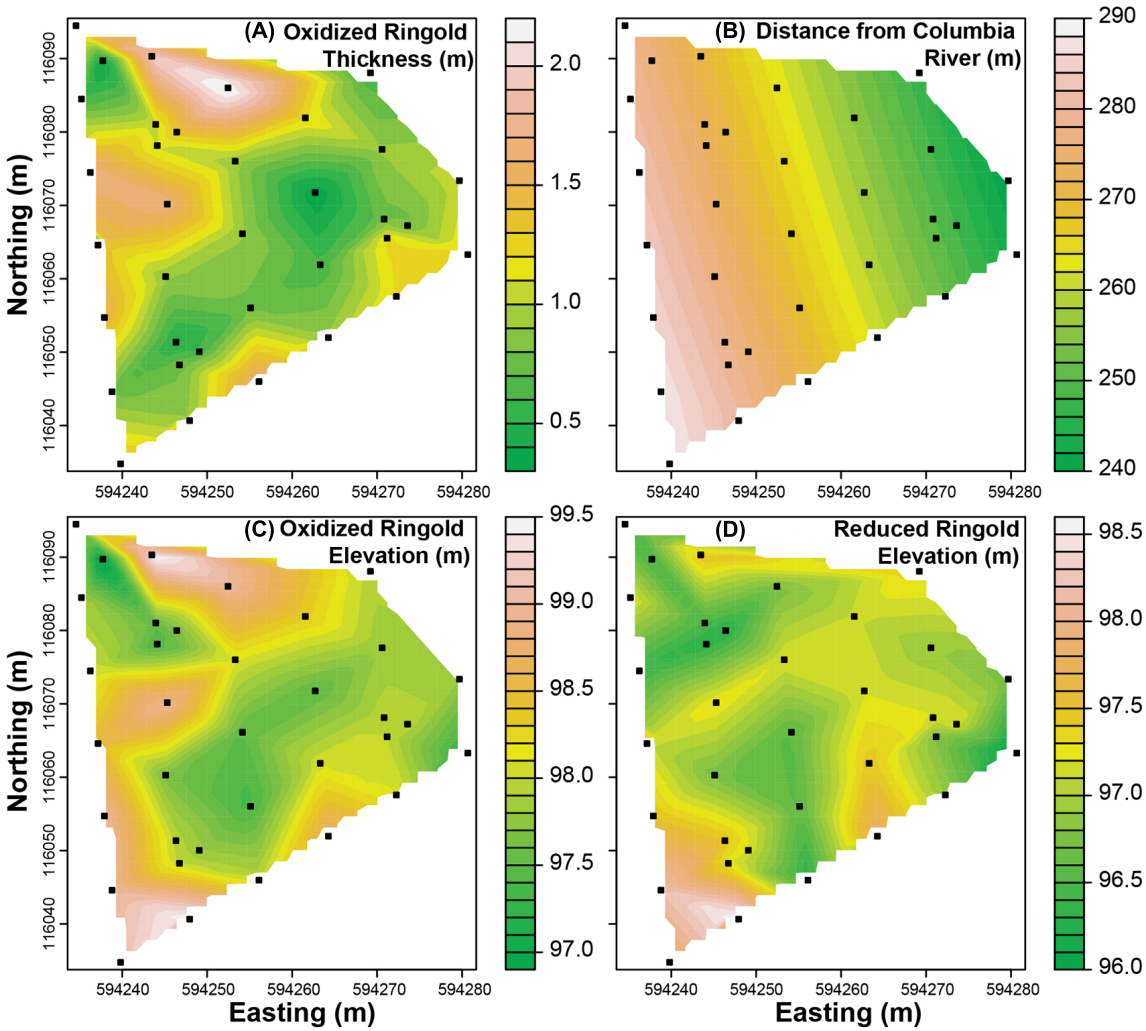
**Maps of measured environmental features evaluated as predictors of estimates of ecological-process-influences. (A)** The vertical thickness of the oxidized portion of the Ringold formation, **(B)** the horizontal distance from the Columbia River, **(C)** the elevation at the top of the oxidized Ringold formation, and **(D)** the elevation at the top of the reduced portion of the Ringold formation. Solid symbols indicate sampling locations and colors indicate interpolations with magnitudes corresponding to scale bars associated with each panel.

**FIGURE 3:**
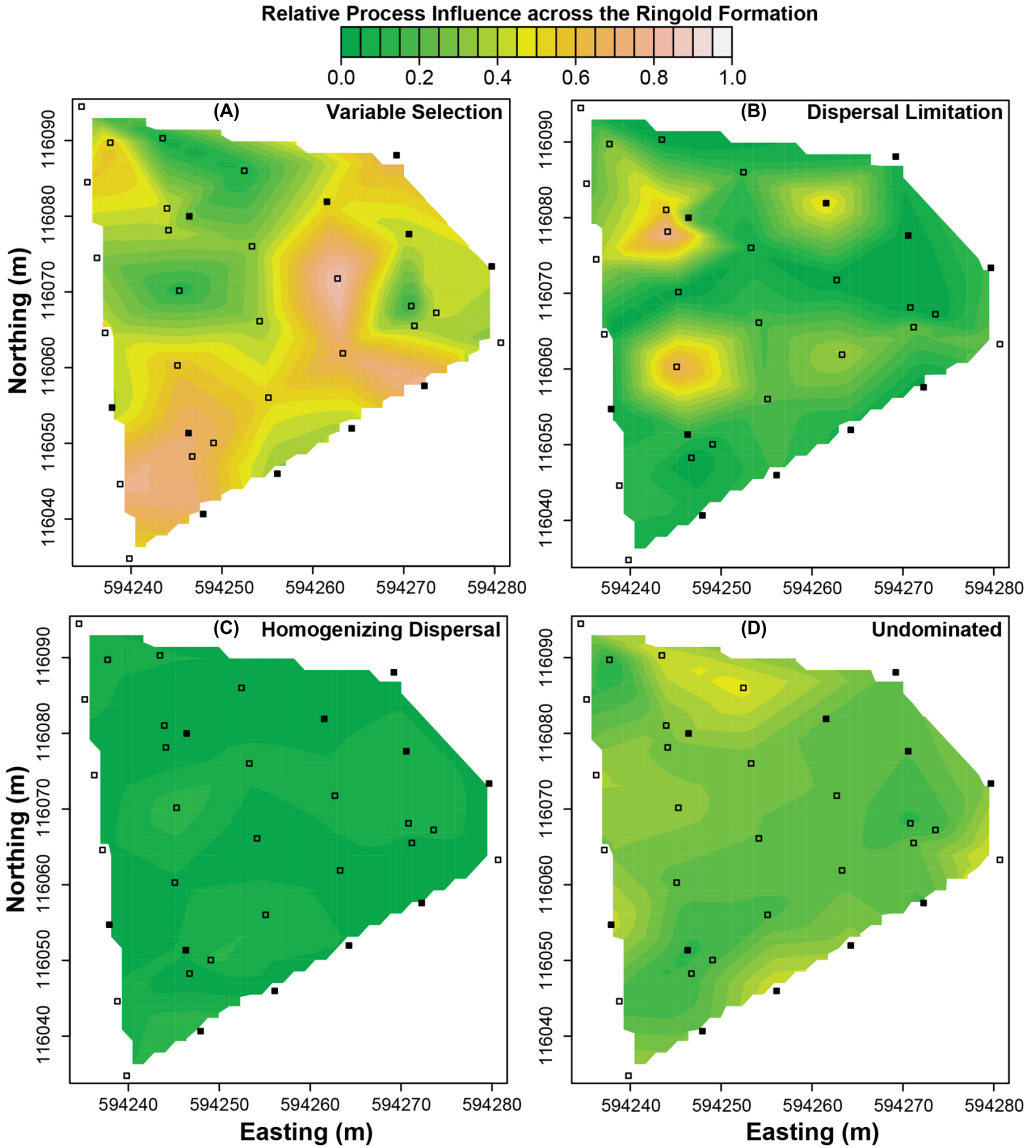
**Predicted spatial variation in the relative influences of ecological processes across the Ringold formation. (A)** Variable selection, **(B)** dispersal limitation, **(C)** homogenizing dispersal, and **(D)** the undominated fraction. Squares indicate spatial locations where field samples were used to estimate environmental features. Filled squares indicate where field samples were also used to characterize microbial communities. The scale bar applies to all panels.

**FIGURE 4:**
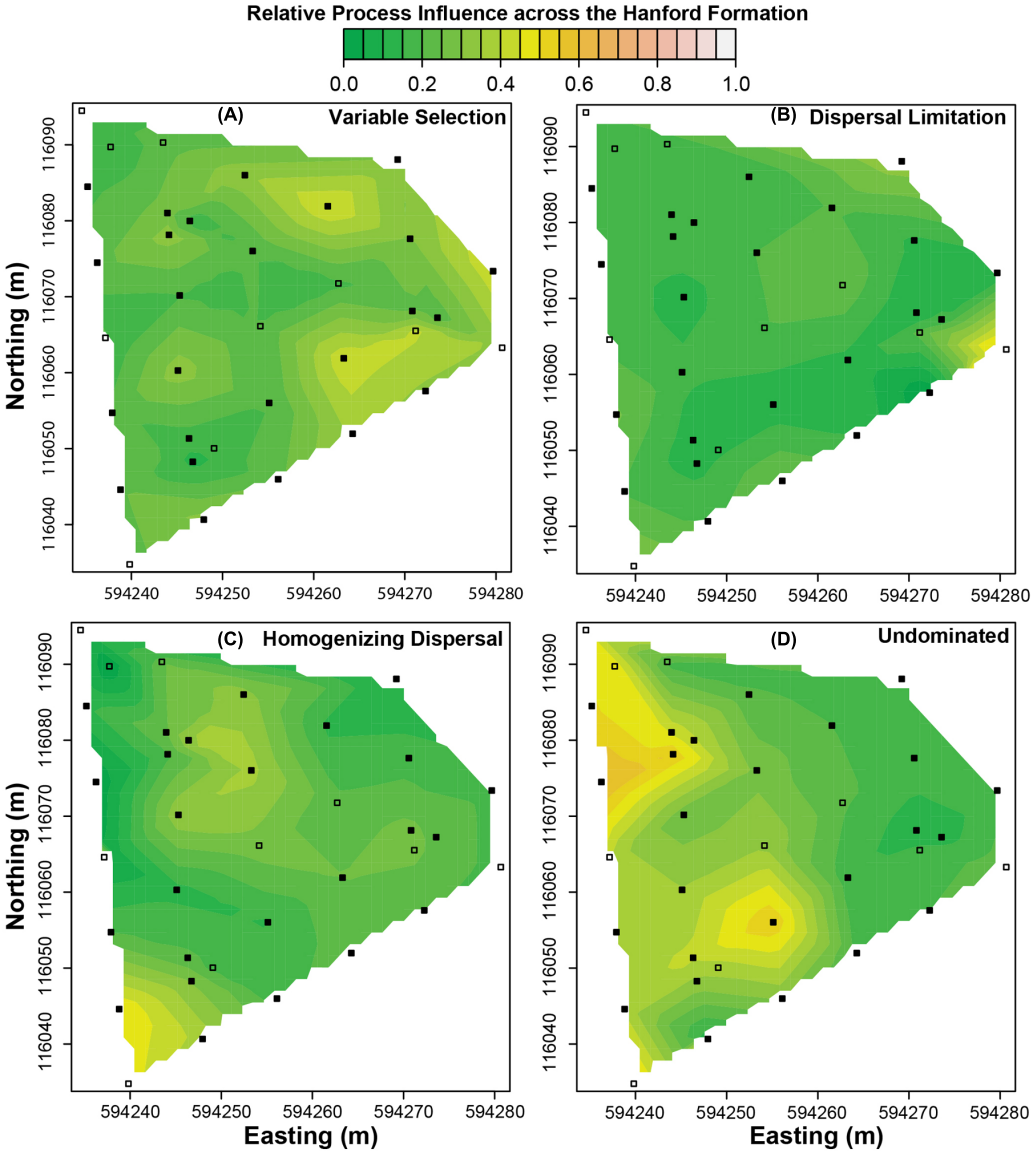
**Predicted spatial variation in the relative influences of ecological processes across the Hanford formation. (A)** Variable selection, **(B)** dispersal limitation, **(C)** homogenizing dispersal, and **(D)** the undominated fraction. Squares indicate spatial locations where field samples were used to estimate environmental features. Filled squares indicate where field samples were also used to characterize microbial communities. The scale bar applies to all panels.

For each ecological process we used environmental features and formation-specific PCNM axes as explanatory variables to construct all possible models with up to seven independent variables. Explanatory variables were described using data from all locations for which there were environmental data (**Figure 2**), but ecological-process estimates were only available from locations where microbial communities were sampled (**Figures 3** and **4**). While three of the environmental variables characterize the Ringold Formation, they were used as potential explanatory variables in models for both formations. Doing so evaluated the hypothesis that features of the Ringold Formation influence ecological processes in one or both formations.

Microbial communities were characterized at 16 and 28 locations in the Ringold and Hanford, respectively. We therefore chose the statistical (i.e., the linear, multiple regression) model with the lowest small-sample-size-corrected Akaike Information Criterion (AICc; Burnham and Anderson, 2002). Prior to the construction of statistical models some explanatory variables were removed to avoid strong co-variation. The set of retained explanatory variables was unique to each ecological process in each formation, and was determined as follows. First, explanatory variables were prioritized; a primary goal was to determine the influence of measured environmental features over spatial patterns in ecological processes; measured environmental features had higher priority than the spatial PCNM axes. In addition, ranking within each class of variable—environmental or PCNM—was based on the strength of correlation to the ecological process being evaluated. If two explanatory variables were significantly correlated (R function ‘cor.test’), only the explanatory variable with higher priority was retained. Using this approach, lower priority variables that were strongly correlated with higher priority variables were removed prior to model selection.

Models with the lowest AICc for each ecological process in each formation were used to construct spatial maps of ecological-process-influences. For each ecological process in each formation, estimates of explanatory variables from each sampled location were fed into the selected models to generate predictions of ecological-process-influences. This was done for all sampled locations, which included locations where microbial communities were characterized and locations where microbial communities were not characterized. The statistical models were therefore used to extrapolate to locations without microbial data. In turn, the predicted ecological-process-influences were spatially interpolated (function ‘interp’ in R library ‘akima’) and visualized (function ‘filled.contour’ in R package ‘graphics’).

To help determine which environmental features are most likely to have a causal effect on ecological-process-influences, environmental features were related to ecological-process-influences using univariate linear regression. This was only done when a measured environmental feature was the most important component within the multiple regression model of a given ecological process; the most important variable had the largest absolute standardized regression parameter.

## Simulation Model

### Overview

The preceding two subsections contain a number of assertions regarding the interpretation of βNTI and RC_bray_ that need to be evaluated to refine interpretations and better understand limitations. For an initial evaluation we developed a purposely simple simulation model that uses idealized cases of specific ecological scenarios to generate a set of ecological communities that are used to evaluate our proposed interpretations.

**FIGURE 5:**
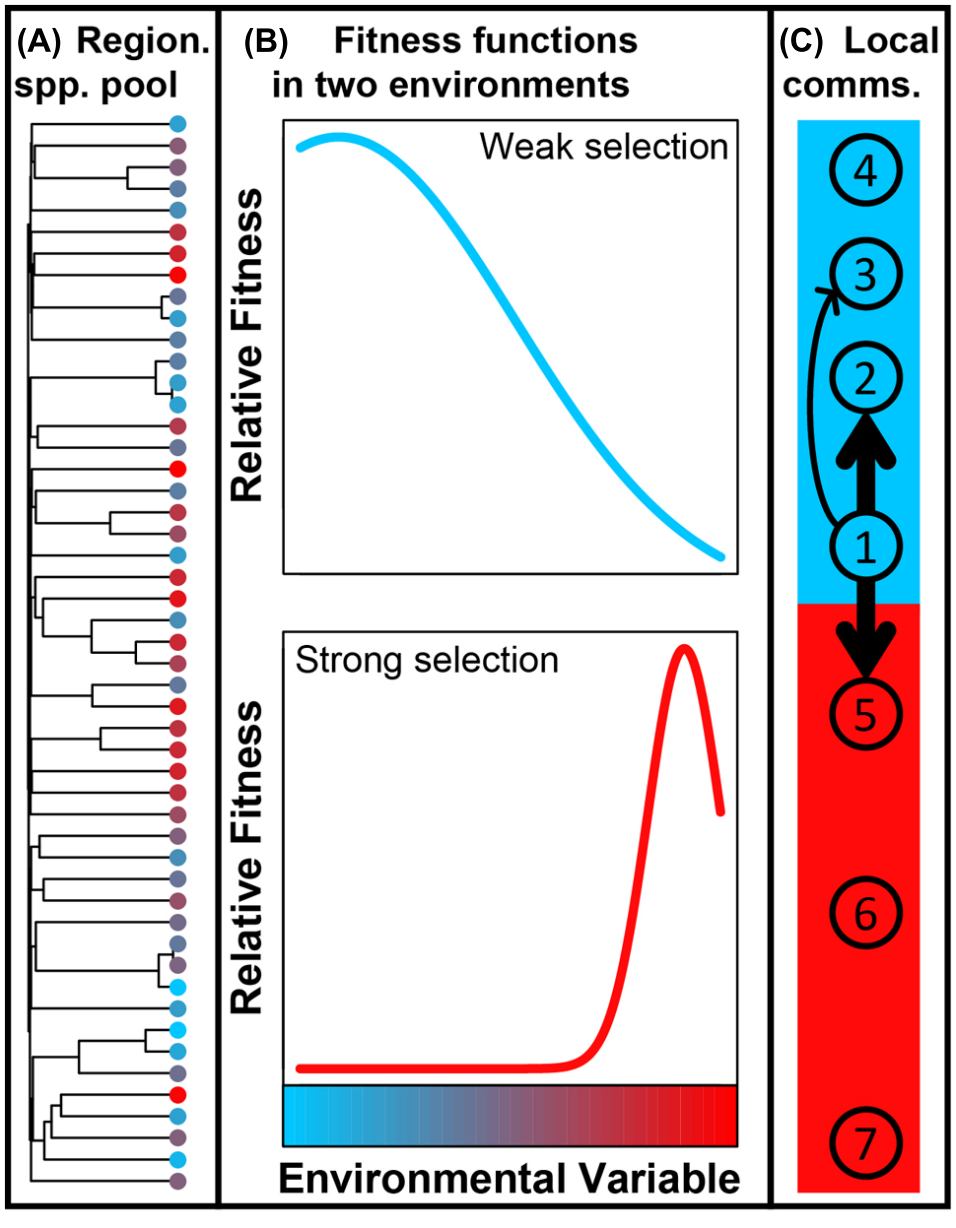
**(A)** Phylogeny showing evolutionary relationships among a subsample of 50 species from the regional species pool simulation model; colors indicate environmental optimum of each species along the arbitrary environmental gradient. **(B)** Fitness functions in two environments along the environmental gradient (see color ramp on bottom panel) that—for illustration—could be cold (blue) and hot (red). The ‘cold’ environment is characterized by relatively weak ecological selection; the blue Gaussian function describes how fitness declines as a species environmental optimum deviates from the blue environment’s temperature. The ‘hot’ environment is characterized by strong selection; fitness declines rapidly as species environmental optima move away from the red environment’s temperature, as indicated by the red Gaussian fitness function. **(C)** Assembly of all local communities was simulated by probabilistic dispersal (not diagrammed) from the regional pool; a species’ probability of dispersal and its local abundance increased with its fit to the local environment. Species abundances in communities 2, 3, and 5 were further influenced by dispersal from community 1; arrows indicate dispersal and thicker arrows indicate higher dispersal rates. Primary and secondary ecological processes governing turnover between pairs of communities—and the associated expectations for βNTI and RC_bray_—are summarized in **Table 1**.

The simulation model has two components; the first evolves a regional pool of species, tracking species’ evolutionary relationships and evolution in species’ optimal environments; the second uses this regional pool to assemble local communities based on scenarios that reflect our conceptualization of how each ecological process influences community composition (**Figure 5**).

The first component of the model simulates diversification in which new species arise asexually through mutations in the environmental optima of ancestral species. Environmental optima evolve and diversify along an arbitrary environmental axis that takes on values from 0 to 1. Evolution is effectively Brownian due to no variation in fitness across the environmental axis. The number of species in the regional pool reaches equilibrium due to the following constraints, which are similar to those imposed in Hurlbert and Stegen (2014): there is a maximum total number of individuals (2 million) summed across all species such that population sizes (equal across species) decline with increasing number of species, and the probability of species extinction increases with decreasing population size following a negative exponential function [population extinction probability ∝ exp(-0.001^∗^population size)].

The system was initiated with one ancestor that had a randomly chosen environmental optimum. The maximum number of individuals in the system was always achieved such that the ancestor had an initial population size of 2 million. The probability of mutation increased with population size, and a descendant’s environmental optimum deviated from its ancestor’s by a quantity selected from a Gaussian distribution with mean of 0 and SD of 0.15. Following mutation, population sizes were adjusted so that the total number of individuals was 2 million. Within a time step, mutation and extinction occurred probabilistically and population sizes were adjusted. The simulation was run for 250 time steps, which was sufficient to reach equilibrium species richness. This simulation procedure, which included tracking of evolutionary relationships among species, provided significant phylogenetic signal (Supplementary Figure S1) for a regional species pool comprised of 1140 species with environmental optima that spanned the environmental axis (Supplementary Figure S2).

In the ecological component of our model, species were drawn from the simulated regional pool to assemble four communities under relatively weak ecological selection (blue environment, **Figure 5**) and three communities under stronger ecological selection (red environment, **Figure 5**). All communities had 100 species and 10,000 individuals, drawn probabilistically from the regional species pool using ecological rules summarized below. The ecological rules enabled development of *a priori* expectations (summarized in **Table 1**) for the magnitudes of βNTI and RC_bray_, under the assumption that greater fitness translates into higher relative abundance. Following community assembly these expectations were evaluated by comparing one focal community to five other simulated communities, with one exception discussed below.

**Table 1:** Ecological processes primarily, and secondarily, responsible for turnover between indicated pairs of communities within the simulation model (****Figure 1**** depicts relationships among communities).

Communities	Primary factor	Primary expectation	Primary supported	Secondary factor	Secondary expectation	Secondary supported	Fraction unexpected
1 and 2	Homogenizing dispersal	| βNTI | < 2 RC_bray_ <-0.95	91%	Homogeneous selection	βNTI < -2	8%	1%
1 and 3	Undominated	| βNTI | < 2 |RC_bray_| < 0.95	87%	Homogeneous selection	βNTI < -2	11%	2%
1 and 4	Dispersal limitation	| βNTI | < 2 RC_bray_ > +0.95	88%	Homogeneous selection	βNTI < -2	12%	1%
1 and 5	Homogenizing dispersal	| βNTI | < 2 RC_bray_ < -0.95	83%	Variable selection	βNTI > +2	16%	1%
1 and (6 or 7)	Variable selection	βNTI > +2	91%	Dispersal limitation	| βNTI | < 2 RC_bray_ > +0.95	9%	0%
6 and 7	Homogeneous selection	βNTI < -2	90%	Dispersal limitation	| βNTI | < 2 RC_bray_ > +0.95	0%	10%

Primary and secondary ecological processes are inputs to the simulation model and the associated expectations are patterns that our statistical framework should reveal if it is properly parsing the influences of those ecological processes. The degree of support is summarized as the percentage of replicate simulations for which the tatistical framework generates the expected magnitudes of βNTI and RC_bray_. The frequency of observing combinations of these two metrics that depart from both the primary and secondary expectations is the scenario-specific error (Fraction unexpected).

To characterize the degree of support for our proposed interpretations of βNTI and RC_bray_, the ecological assembly model was run 1000 times. Each iteration used the same regional pool, which was evolved once, and βNTI and RC_bray_ were quantified following community assembly. This approach generated a distribution of both metrics for each pairwise community comparison. These distributions were used to characterize the degree of support for our *a priori* expectations, which were tied directly to our proposed conceptual interpretations of βNTI and RC_bray_. We next describe the different ecological rules imposed within the assembly model.

### The Focal Community (Community 1)

Hundred species were drawn without replacement from the regional species pool with probabilities proportional to their fitness in an environment of 0.05; fit was quantified with a Gaussian function centered on 0.05 and with variance of 0.175 (blue curve, **Figure 5**); the arbitrary environmental variable used in the simulations took on values between 0 and 1. Individuals were probabilistically drawn into the 100 selected species until reaching 10,000 individuals. Note that the strength of selection is relatively weak in the blue environment as indicated by the relatively broad fitness function; for comparison, note that selection is relatively strong in the red environment as indicated by the narrower fitness function (**Figure 5**).

### Homogenizing Dispersal (Community 2)

Species and individuals were probabilistically drawn as for community 1, but the probabilities were altered to reflect a high rate of dispersal from community 1. More specifically, species’ probabilities based on the Gaussian fitness function (within the blue environment) were modified by adding a quantity equal to 0.05^∗^(species abundance in the focal community ^1.1^); the exponent controls the rate of dispersal and was selected through preliminary exploration of parameter-space. Given a high rate of dispersal between communities 1 and 2, compositional turnover between communities 1 and 2 will be primarily governed by homogenizing dispersal; the expectations are |βNTI| < 2 and RC_bray_ < -0.95.

### Undominated (Community 3)

In our conceptualization, an ‘undominated’ scenario arises when there is a moderate rate of dispersal and the strength of selection is relatively weak; high dispersal leads to homogenizing dispersal, low dispersal leads to dispersal limitation, and strong selection will constrain community composition. To generate this scenario, species were drawn into community 3 as for community 2, but with a lower dispersal rate; species’ probabilities based on the Gaussian fitness function were modified by adding a quantity equal to 0.05^∗^(species abundance in the focal community^0.8^); this smaller exponent caused a lower rate of dispersal. Compositional turnover between communities 2 and 3 should be undominated such that |βNTI| < 2 and | RC_bray_| < 0.95 are expected.

### Dispersal Limitation (Community 4)

Species were drawn into community 4 using only probabilities based on fit to the environment (as for community 1 assembly). This reflects dispersal limitation in the sense that the abundance of a given species in community 1 had no influence on its abundance in community 4 because there is no direct dispersal between the communities. Given weak selection in the blue environment and a low dispersal rate between communities 1 and 4, compositional turnover between communities 1 and 4 should be dominated by dispersal limitation; |βNTI|< 2 and RC_bray_ > +0.95 are expected.

### Homogenizing Dispersal Overwhelms Variable Selection (Community 5)

To generate this scenario a second selective environment was required, and was represented as the red environment in **Figure 5**, (environmental value of 0.95). Community 5 was assembled in the red environment such that species’ probabilities of occurrence were influenced by their fitness as determined by a Gaussian fitness function centered on 0.95 and with a variance of 0.0075 (red curve, **Figure 5**). The red Gaussian fitness function had lower variance than the blue Gaussian fitness function, which reflected stronger selection. To draw species and individuals into community 5, the fitness-based probabilities were modified as for community 2 assembly such that there was a high rate of dispersal from community 1 to community 5; species’ probabilities based on the Gaussian fitness function (within the red environment) were modified by adding a quantity equal to 0.05*(species abundance in the focal community^1.1^). Given a high rate of dispersal between communities 1 and 5, compositional turnover between communities 1 and 5 should be governed by homogenizing dispersal even though the selective environments are different; |βNTI| < 2 and RC_bray_ < -0.95 are expected.

### Variable Selection (Community 6)

Community 6 was assembled as for community 1, but instead using species’ probabilities based on fitness within the red environment. In this case, large compositional differences should arise between communities 1 and 6 due to these communities being assembled in different selective environments; variable selection should dominate such that βNTI > +2 is expected.

### Homogeneous Selection (Community 7)

Homogeneous selection can dominate if communities occur in the same selective environment and if selection is relatively strong. Ecological selection in the blue environment is relatively weak such that homogeneous selection is unlikely to arise; as selection becomes weaker, species become demographically equivalent so that selection does not govern community composition (as in Hubbell, 2001). Selection in the red environment is relatively strong, however, such that homogeneous selection will emerge when assembly is governed principally by environmentally determined fitness. Community 7 was therefore assembled as for community 6; βNTI < -2 is expected.

### Secondary Expectations

As summarized above, our proposed conceptual interpretations of βNTI and RC_bray_ provide *a priori* expectations for patterns of these metrics in each simulated scenario (**Table 1**). Ecological systems are, however, inherently probabilistic (as is our simulation model). While there is a high probability that species in community (1) disperse to community (2), for example, this is not guaranteed to occur—in the model or in natural systems—and it is therefore expected that homogenizing dispersal will not always dominate. Given that communities (1) and (2) are assembled under the same selective environment, we suggest that when homogenizing dispersal fails to dominate it is most likely because homogeneous selection has constrained community composition; on average, selection is relatively weak in the blue environment (**Figure 5**), but occasionally selection will strongly constrain community composition. The comparison between communities (1) and (2) in a relatively small fraction of replicate simulations should therefore be characterized by βNTI < -2; we consider this to be a ‘secondary expectation.’ For each pairwise community comparison we derived secondary expectations, which are summarized in **Table 1**. The percentage of replicate simulations showing patterns of βNTI and RC_bray_ consistent with those expectations are summarized in **Table 1**.

## Results

Analysis of simulation model outputs showed strong correspondence between expected and observed patterns of βNTI and RC_bray_ (**Table 1**). Five of the six scenarios showing error rates of 2% or less. The scenario with the highest error rate (10%) invoked strong homogeneous selection and was associated with the comparison between communities (6) and (7). The statistical framework was therefore applied to the data on microbial communities across the Hanford and Ringold formations.

In both geologic formations there was substantial variation across local communities in the relative influences of the ecological processes (Supplementary Figure S3). Using environmental features and PCNM axes to describe spatial variation in ecological-process-influences showed that each process within each formation was associated with a distinct set of features/axes (**Tables 2** and **3**). In most cases the best models were highly significant, contained at least one environmental feature, and explained up to 83% of spatial variation in ecological-process-influences (**Tables 2** and **3**).

**Table 2:** Chosen models for each ecological process within the Ringold Formation.

Variable	Coefficient	SE	*t*-value	*p*-value
**Ringold homogeneous selection**: model *R*^2^ = 0.17, *p* = 0.11
PCNM22	0.023	0.014	1.694	0.112
**Ringold variable selection**: model *R*^2^ = 0.81, *p* = 0.0007
Oxidized Ringold Thick	-0.164	0.035	-4.637	0.001
PCNM21	0.103	0.026	3.955	0.002
PCNM2	0.065	0.029	2.241	0.047
PCNM8	0.093	0.034	2.710	0.020
**Ringold undominated**: model *R*^2^ = 0.3847, *p* = 0.04257
Oxidized Ringold Thick	0.088	0.039	2.258	0.042
PCNM17	0.063	0.033	1.893	0.081
**Ringold dispersal limitation**: model *R*^2^ = 0.8298, *p* = 0.001333
Oxidized Ringold Elevation	-0.196	0.038	-5.228	0.0004
PCNM22	-0.176	0.026	-6.802	0.0000
PCNM1	0.125	0.025	4.944	0.0006
PCNM13	-0.039	0.015	-2.669	0.0235
PCNM8	0.171	0.036	4.790	0.0007
**Ringold homogenizing dispersal**: model *R*^2^ = 0.7029, *p* = 0.001742
Reduced Ring. Elevation	0.044	0.009	4.911	0.0004
PCNM8	-0.034	0.010	-3.378	0.0055
PCNM16	-0.033	0.010	-3.449	0.0048

All explanatory variables were standardized as standard normal deviates prior to analysis such that coefficient magnitudes are directly comparable.

**Table 3:** Chosen models for each ecological process within the Hanford formation.

Variable	Coefficient	SE	*t*-value	*p*-value
**Hanford homogeneous selection**: model *R*^2^ = 0.65, *p* = 0.0002
Distance to River	-0.013	0.007	-1.787	0.0877
Oxidized Ringold Thick.	-0.015	0.008	-1.957	0.0632
PCNM22	0.019	0.006	3.023	0.0063
PCNM6	0.019	0.009	2.138	0.0439
PCNM21	0.013	0.007	1.903	0.0702
**Hanford variable selection**: model *R*^2^ = 0.29, *p* = 0.04
Distance to River	-0.061	0.027	-2.252	0.0337
PCNM22	-0.058	0.025	-2.269	0.0325
PCNM8	0.049	0.031	1.611	0.1204
**Hanford undominated**: model *R*^2^ = 0.5271, *p* = 0.0003783
Distance to River	0.114	0.025	4.558	0.0001
Reduced Ring. Elevation	-0.075	0.028	-2.686	0.0129
PCNM11	-0.070	0.027	-2.572	0.0167
**Hanford dispersal limitation**: model *R*^2^ = 0.3908, *p* = 0.00696
PCNM17	0.067	0.028	2.425	0.023
PCNM7	-0.042	0.023	-1.801	0.084
PCNM13	-0.044	0.024	-1.844	0.078
**Hanford homogenizing dispersal**: model *R*^2^ = 0.6128, *p* = 3.664e-05
Oxidized Ringold Elevation	0.042	0.026	1.625	0.1172
PCNM10	-0.075	0.018	-4.103	0.0004
PCNM4	0.069	0.021	3.275	0.0032

All explanatory variables were standardized as standard normal deviates prior to analysis such that coefficient magnitudes are directly comparable.

**FIGURE 6:**
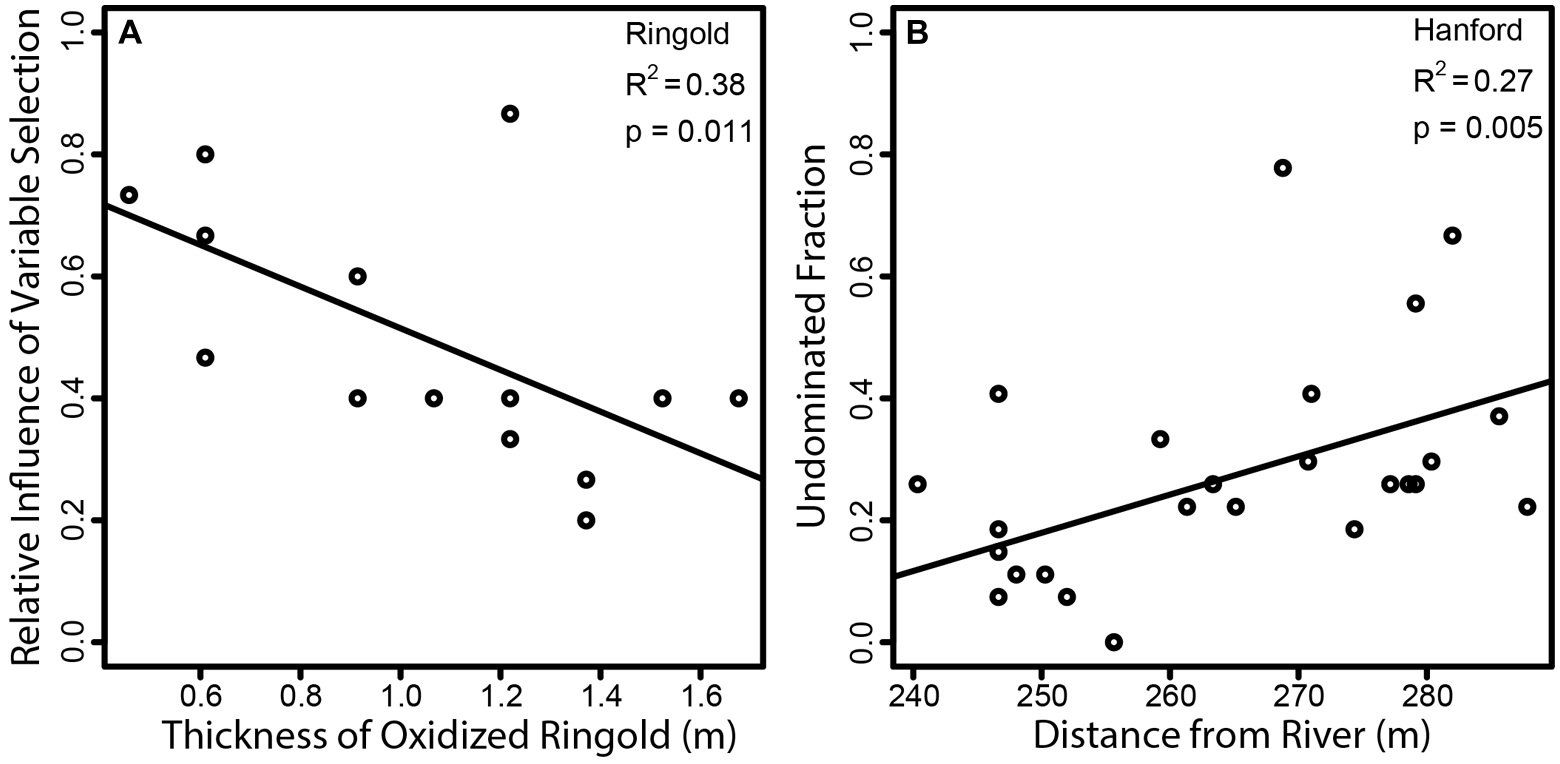
**Regressions of ecological-process-influences against environmental features that were significant in both multiple regression and univariate regression models. (A)** The relationship-within the Ringold formation-between the relative influence of variable selection and the vertical thickness of the oxidized portion of the Ringold formation, and **(B)** the relationship-within the Hanford formation-between the relative contribution of the undominated fraction and horizontal distance from the Columbia River. Solid lines indicate the ordinary least squares regression model, and the geological formation (Ringold or Hanford) and model statistics are provided on each panel.

Within the Ringold Formation, the model for homogeneous selection was not significant (*p* = 0.11), but the thickness of the oxidized Ringold was the most important feature retained in the model for variable selection (**Table 2**). A significant relationship between the influence of variable selection and the thickness of the oxidized Ringold was also observed by univariate regression (*p* = 0.01, *R*^2^ = 0.38, **Figure 6A**). Oxidized Ringold thickness was also the most important feature retained in the model of the undominated fraction (**Table 2**), but this relationship was not significant when evaluated by univariate regression (*p* = 0.07). The upper elevation of the oxidized Ringold was the most important feature retained in the model of dispersal limitation (**Table 2**), but this relationship was not significant when evaluated by univariate regression (*p* = 0.75). The elevation at the top of the reduced Ringold was the most important feature in the homogenizing dispersal model (**Table 2**), but this relationship was not significant when evaluated with univariate regression (*p* = 0.09).

Within the Hanford formation, homogeneous selection was most strongly related to PCNM axes and variable selection was most strongly related to distance from the Columbia River (**Table 3**). Variable selection in the Hanford was not, however, significantly related to distance from the Columbia River, by univariate regression (*p* = 0.12). Distance from the Columbia River was the most important feature in the model of the undominated fraction (**Table 3**), and this relationship was also observed by univariate regression (*p* = 0.005, *R*^2^ = 0.27, **Figure 6B**). No environmental features were retained in the dispersal limitation model (**Table 3**). Homogenizing dispersal was most strongly related to PCNM axes (**Table 3**).

In the Ringold, process-influence-maps revealed spatial patterns of variable selection and in the undominated fraction (**Figure 3**) that showed similarities to spatial patterns of oxidized Ringold thickness (cf. **Figures 2A** and **3A,D**). Dispersal limitation also showed marked spatial variation across the Ringold Formation, with some congruence with spatial patterns in the elevation of the oxidized Ringold (cf. **Figures 2C** and **3B**). In contrast, homogenizing dispersal was characterized by relatively little spatial variation across the Ringold Formation (**Figure 3C**). The model for homogeneous selection was not significant within the Ringold Formation (see above) such that a map was not generated and, in turn, spatial variation was not evaluated.

Process-influence-maps in the Hanford revealed increases in variable selection and decreases in the undominated fraction in regions closest to the Columbia River and complex patterns across regions further from the river (cf. **Figures 2B** and **4A,D**). Homogeneous selection was relatively consistent through space (Supplementary Figure S4). The influence of dispersal limitation in the Hanford appears to be greatest near the eastern corner of the investigated spatial domain (**Figure 4B**), with no obvious correspondence to environmental features, which is consistent with no environmental variables being retained in the associated multiple regression model (**Table 3**). Homogenizing dispersal across the Hanford was characterized by a complex spatial pattern without any obvious correspondence to environmental features.

## Discussion

Here we worked to improve understanding of the ecological processes that influence microbial community composition. A major component of our approach was extending the statistical framework developed in Stegen et al. (2013), which generated process estimates at the scale of a metacommunity and lumped variable and homogeneous selection into a single estimate. Our extension of their framework distinguished homogeneous selection from variable selection and estimated the influences of ecological processes for local communities. This extended framework revealed a dominant influence of variable selection—relative to homogenous selection—and enabled an evaluation of subsurface environmental features related to ecological-process-influences. In turn, we generated process-influence-maps across two geologic formations. Also distinct from Stegen et al. (2013), we evaluated the statistical framework via simulation, which showed close correspondence between expected and observed patterns of βNTI and RC_bray_ (**Table 1**). This provides confidence that our statistical framework generates reasonable estimates of ecological-process-influences.

The comparison between communities 6 and 7—in which homogeneous selection was invoked—resulted in the highest error rate (10%). We hypothesize that increasing the strength of selection would reduce this error rate. Simulation studies that continuously vary the strength of selection could be used to evaluate this hypothesis. Such studies could also be used to go beyond the discrete ecological scenarios studied here. These scenarios were used to enable a tractable evaluation of our approach to inferring ecological processes from null modeling results, but selection strength and dispersal rate are continuous variables (e.g., Stegen and Hurlbert, 2011). If our approach is robust, null modeling results should change continuously with the strength of selection and rate of dispersal. Follow-on simulation studies will be needed to fully understand this coupling.

Direct comparison between our results and those from other systems is not currently feasible; we are not aware of previous work that parses homogeneous selection, variable selection, dispersal limitation, and homogenizing dispersal. We note, however, that our results are consistent with dispersal limitation having an important influence over microbial community composition. This aligns with the emerging perspective that microbes have biogeography (Green et al., 2004, 2008; Martiny et al., 2006) and suggests that in the subsurface all microbes are not everywhere, in contrast to the classic perspective (De Wit and Bouvier, 2006; Martiny et al., 2006).

### Conceptual Inferences

In both formations variable selection and the undominated fraction showed opposing, spatially structured patterns while dispersal limitation and homogenizing dispersal showed more idiosyncratic patterns. To a first approximation variable selection in the Ringold was maximized along a Southwest to Northeast axis, but was maximized in the Eastern corner of the Hanford formation. Spatial patterns of dispersal limitation and homogenizing dispersal were also different between formations. Environmental features governing the relative influences of ecological processes therefore appear to be formation-specific.

The influence of variable selection in the Ringold was most strongly related to oxidized Ringold thickness; variable selection became increasingly weak with increasing thickness (**Figure 6A**). If one defines the rate at which redox conditions change with depth as the vertical distance between oxidized and reduced conditions, redox conditions must change more rapidly with depth in locations with a thinner oxidized Ringold layer. In turn, our results suggest that variable selection increases as the vertical redox gradient becomes steeper (i.e., when redox conditions change more rapidly with depth).

The influence of variable selection may increase with steeper redox gradients if a small number of microbial taxa take advantage of rapidly changing redox conditions. This hypothesis could be directly tested by evaluating the response of microbial communities to experimentally manipulated redox patterns within laboratory flow cells. Such an approach would leverage the strengths of comparative and experimental techniques to provide a deeper level of understanding than otherwise possible (Weber and Agrawal, 2012).

In the Hanford formation the undominated fraction decreased toward the Columbia River (**Figure 4D**; **Table 3**), and there was a modest increase in the influence of variable selection toward the Columbia River (**Figure 4A**; **Table 3**). These patterns suggest an important environmental shift as one moves toward the river, which may be related to spatially structured river-water intrusion.

Elevation of the Columbia River increases annually in the spring, causing river-water intrusion into the studied subsurface system (Peterson et al., 2008; Lin et al., 2012b). To a first approximation, regions of the system that are closer to the river (**Figure 2B**) receive more river-water (Lin et al., 2012b). It may be that an annually repeated pattern of more river-water in the eastern portion of the Hanford formation has resulted in a shift in environmental conditions that cause a stronger influence of variable selection in that region. As a first test of this hypothesis it would be useful to sample a broader spatial domain that contains regions of the subsurface closer to and further from the river, relative to what has been sampled here. Coupling this expanded sampling with laboratory flow cell experiments would provide powerful hypothesis tests.

The undominated fraction should increase with decreases in the strength of selection and/or with a shift toward moderate dispersal rates. In both formations the undominated fraction generally showed patterns opposite those of variable selection. This suggests that a shift toward undominated compositional turnover is due primarily to weaker selection, as opposed to a change in dispersal rates. It is also interesting to note that both formations were characterized by relatively weak influences of homogeneous selection. This result is expected as selection is unlikely to be consistent across spatially heterogeneous systems such as the subsurface system studied here.

Dispersal limitation and homogenizing dispersal both have complex spatial structure, and it is difficult to discern which environmental features (if any) drive these spatial patterns. Dispersal limitation across both formations was unrelated to environmental features when evaluated with univariate regression, suggesting that the environmental features used in our analyses do not strongly determine the influence of dispersal limitation. Similarly, homogenizing dispersal in the Hanford was more strongly related to PCNM axes than to environmental features (**Table 3**). Instead of being driven by relatively simple environmental features, the influences of dispersal limitation and homogenizing dispersal may be governed more by complex and spatially inconsistent features that influence hydrologic transport.

### Comparison to Variation Partitioning

While it may appear that variation partitioning (Legendre and Legendre, 1998) was used here to estimate ecological-process-influences, there are substantial differences between variation partitioning and our approach. In variation partitioning, variation in community composition is explained using features deemed *a priori* to reflect spatial relationships or environmental differences among communities (e.g., Tuomisto et al., 2003; Cottenie, 2005; Legendre et al., 2009; Heino et al., 2011). Intuitively, the more variation in community composition explained by spatial or environmental features, the greater the influence of dispersal limitation or selection, respectively (Stegen and Hurlbert, 2011). At least three studies have shown that this intuitive expectation is not valid and that variation partitioning cannot be used to infer the influences of ecological processes (Gilbert and Bennett, 2010; Smith and Lundholm, 2010; Stegen and Hurlbert, 2011). Variation partitioning can, at best, determine whether dispersal limitation has more or less influence than selection (Stegen and Hurlbert, 2011).

With variation partitioning it is tempting to infer that variation in community composition not explained by spatial or environmental features results from ecological drift. Unexplained variation will, however, increase artificially if any of the following occur: (*i*) important environmental features have not been measured; (*ii*) spatial axes fail to capture idiosyncratic patterns in spatial isolation among communities; or (*iii*) community composition is non-linearly related to explanatory variables (see discussions in Laliberte et al., 2009; Legendre et al., 2009; Anderson et al., 2011).

In contrast to variation partitioning we used null models to estimate the influences of ecological processes; our approach *does not* relate community composition to explanatory variables. Our approach therefore solves a primary shortcoming of variation partitioning, but like all statistical frameworks has its own limitations.

### Limitations, Caveats, and the Path Forward

With respect to the framework used here, two important considerations are sample size and error from sampling, DNA sequencing and data processing. Ecological process estimates likely approach their true values as sample size increases and this should be considered in cross-system comparisons.

It is not obvious that sources of error will systematically increase or decrease any particular process estimate such that we assume errors contribute equally to estimates of all ecological process. Nonetheless, future simulation studies need to evaluate the potential for bias related to sample size and sources of error.

On the conceptual side, we note that our framework does not account for influences of *in situ* diversification, which is the evolutionary component in Vellend’s (2010) synthesis (see also Hanson et al., 2012). This may be relevant as high rates of *in situ* diversification could increase compositional turnover in terms of OTU presence/absence; if dispersal rates are low, new OTUs that evolve *in situ* may only be found in one community. On the other hand, among-community dispersal will minimize influences of *in situ* diversification; if newly evolved OTUs disperse away from their community-of-origin, the influence of *in situ* diversification on compositional turnover will be minimal. Outcomes are therefore influenced by the balance between dispersal and diversification rates.

Extending our framework to characterize the influence of *in situ* diversification on patterns of compositional turnover represents an important challenge. Pattern-oriented simulation modeling (e.g., Grimm et al., 2005; Rangel et al., 2007; Stegen and Hurlbert, 2011; McClain et al., 2012) is one approach that could be leveraged to meet this challenge. In the context of species richness gradients, for example, Hurlbert and Stegen (2014) recently developed a pattern-oriented simulation model that provides ‘multi-metric fingerprints’ that indicate the operation of specific underlying processes. A similar approach could be added to our framework to identify multi-metric fingerprints indicating the influence of *in situ* diversification on patterns of compositional turnover.

We further suggest that a pattern-oriented simulation modeling approach could be used to draw additional inferences from the undominated fraction provided by our current framework. The undominated fraction is tied to a conceptual inference—selection is too weak and dispersal rates are not extreme enough for either process to drive compositional turnover. It would be useful, however, to parse the relative influences of ecological processes within the undominated fraction. To this end, pattern-oriented simulations similar to those in Stegen and Hurlbert (2011) could be used to study compositional turnover patterns expected across a two-dimensional process-space defined by the strength of selection and the rate of dispersal. Observed turnover patterns within the undominated fraction could then be related back to simulated turnover patterns and, in turn, the balance between selection and dispersal (similar to McClain et al., 2012).

In summary, we have provided spatial projections for the relative influences of ecological processes on subsurface microbial community composition. Doing so has revealed key features of our system unrecognized through application of existing statistical frameworks. We suggest that for many systems a similar outcome is likely and that novel insights can be gained through broad application of a framework that couples the approach used here with that of Stegen et al. (2013). We look forward to these statistical frameworks being improved through additional simulation-based evaluations, to experimental tests of the hypotheses they generate, and to a coupling between the information they provide and process-based models aimed at predicting community composition across environmental conditions.

## Conflict of Interest Statement

The authors declare that the research was conducted in the absence of any commercial or financial relationships that could be construed as a potential conflict of interest.

## Footnotes

1 http://ifchanford.pnnl.gov/

2 http://cran.r-project.org/
