# Supplementary material for: Estimating and mapping ecological processes influencing microbial community assembly
Source: Front Microbiol. 2015 May 1;6:370. doi: 10.3389/fmicb.2015.00370 (PMC4416444; doi:10.3389/fmicb.2015.00370)
Supplement: Supplementary file 1 [file Presentation_1.PDF]

Supplementary Material

Estimating and Mapping Ecological Processes Influencing Microbial Community Assembly

James C. Stegen[1]*, Xueju Lin[1], Jim K. Fredrickson[1], Allan E. Konopka[1]

[1]Fundamental and Computational Sciences Directorate, Biological Sciences Division, Pacific Northwest National Laboratory, Richland, WA, USA

*** Correspondence:** James C. Stegen, Fundamental and Computational Sciences Directorate, Biological Sciences Division, Pacific Northwest National Laboratory, Richland, WA, USA
James.Stegen@pnnl.gov

1. Supplementary Figures and Tables

1.1. Supplementary Figures

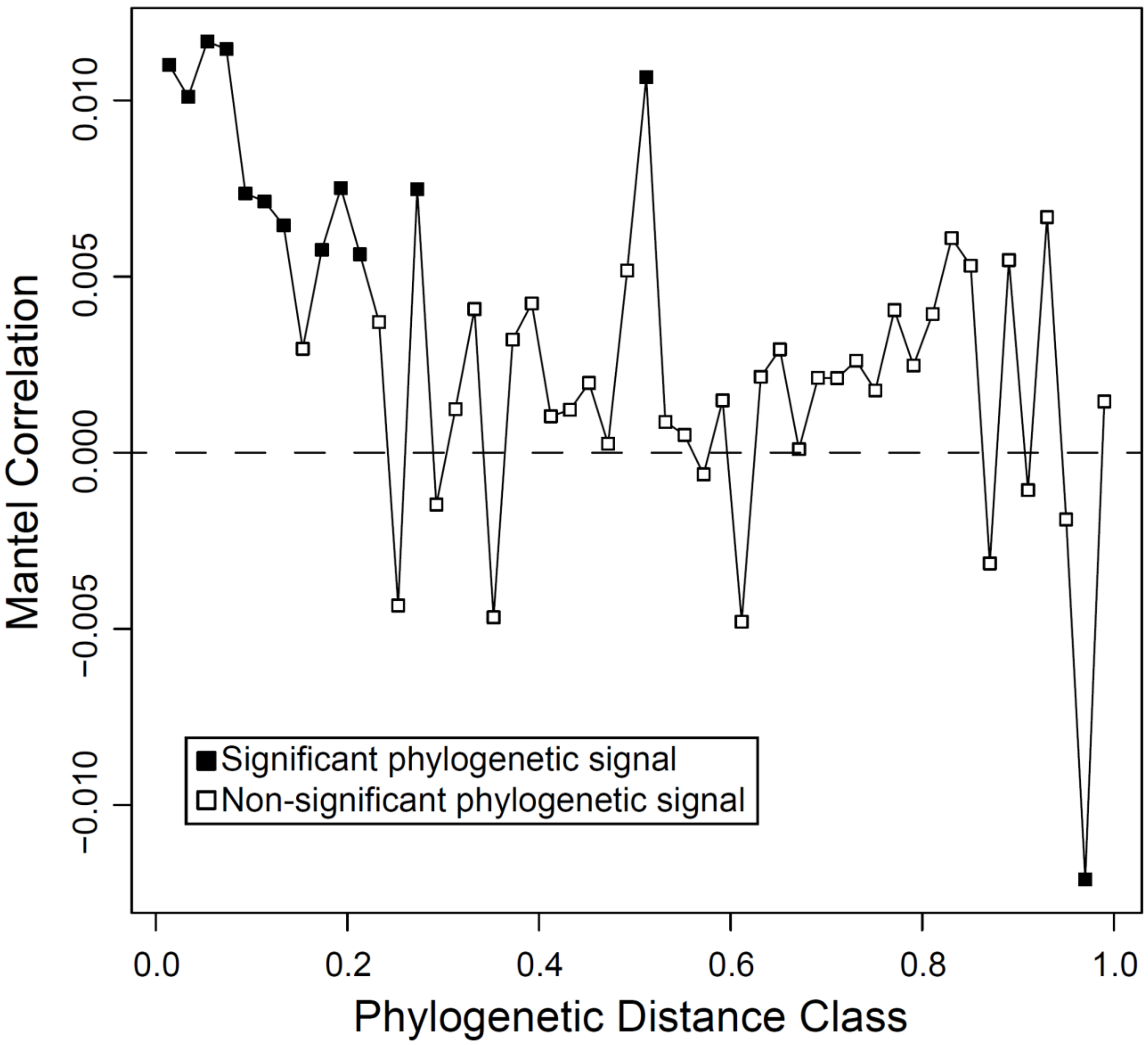

Supplementary Figure 1. Phylogenetic Mantel correlogram indicating significant phylogenetic signal across relatively short phylogenetic distances, arising from the regional-species-pool-evolution simulation model. Correlations are derived from relating between-species differences in environmental optima to between-species phylogenetic distances. Significantly positive correlations (solid squares) indicate that environmental-optima-distances between species increase with between-species phylogenetic distance, but only across the phylogenetic distance class being evaluated. Significance tests were performed as in Stegen et al. (2013) used 999 permutations in the R function 'mantel.correlog' (package 'vegan') with a progressive Bonferroni correction (Legendre and Legendre, 1998) and no distance class cutoff.

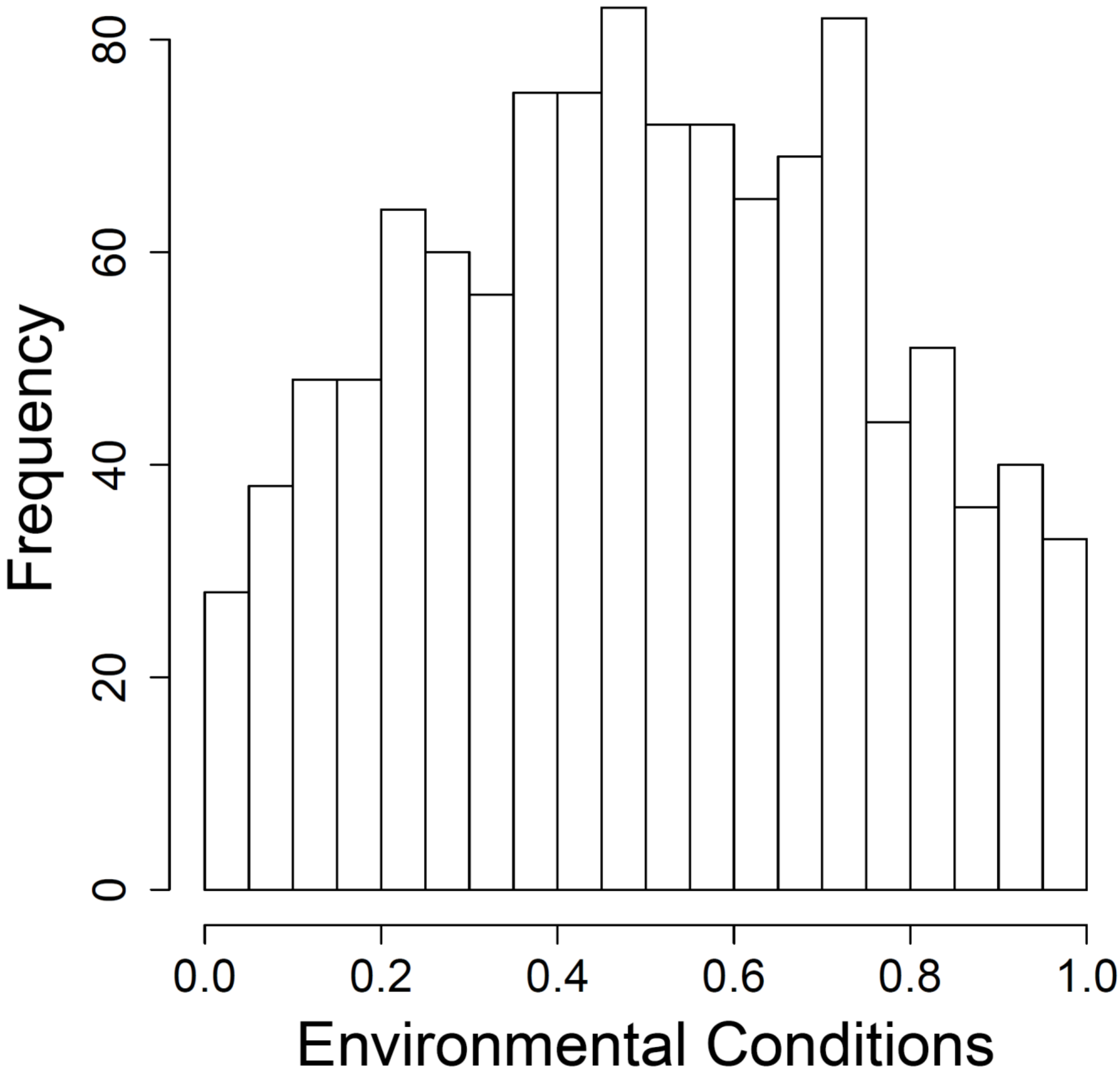

Supplementary Figure 2. Histogram of species' environmental optima arising from the regional-species-pool-evolution simulation model. The environmental conditions represent an arbitrary environmental axis with values ranging from 0 to 1. Fitness is equal across the environmental axis such that evolution of environmental optima is effectively Brownian (i.e., evolution is not directional).

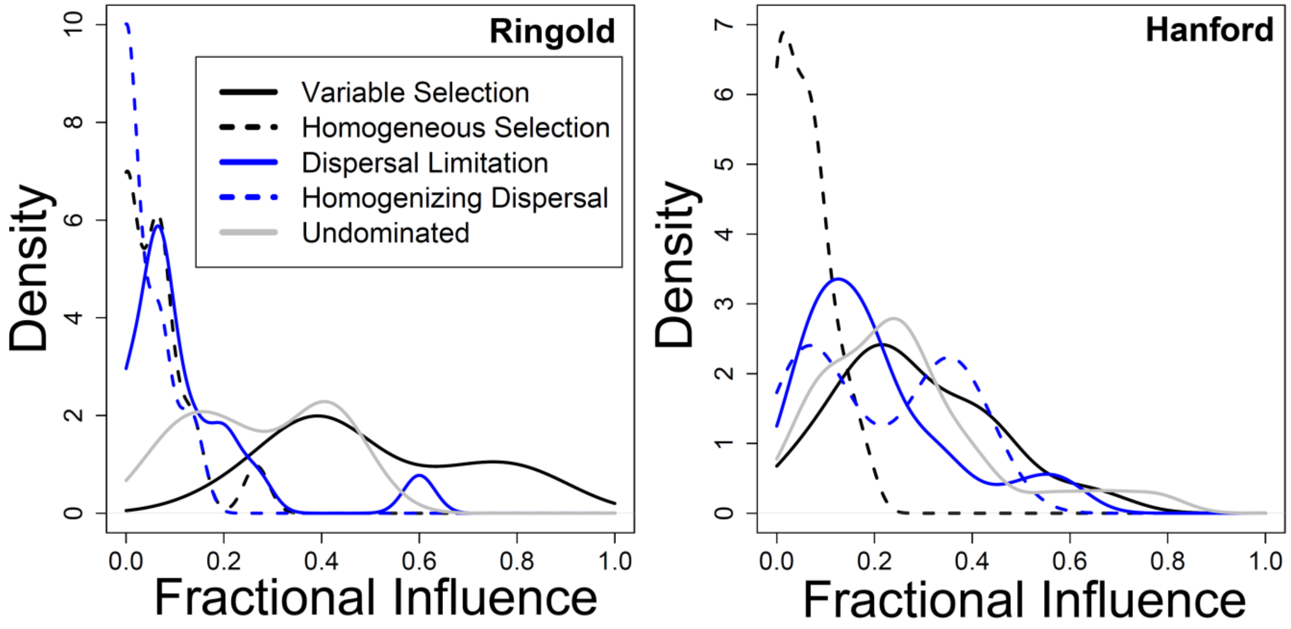

Supplementary Figure 3. Distributions for the relative contributions of variable selection, homogeneous selection, dispersal limitation, homogenizing dispersal, and the undominated fraction in the Ringold and Hanford geologic formations. Data for all communities are included in the distributions, which have been smoothed to facilitate comparison.

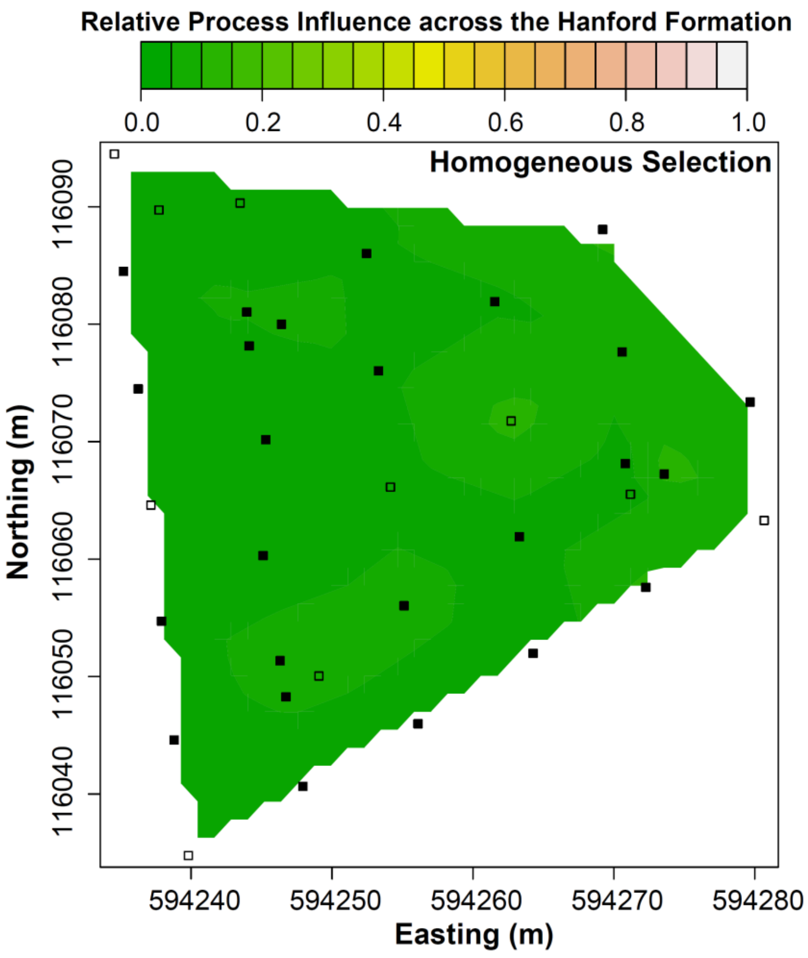

Supplementary Figure 4. Predicted spatial variation in the relative influence of homogeneous selection across the Hanford formation. Squares indicate spatial locations where field samples were used to estimate environmental features. Filled squares indicate where field samples were also used to characterize microbial communities. A multiple regression model attempting to explain spatial variation in homogeneous selection across the Ringold formation was not significant; a map of homogeneous selection could not, therefore, be generated for the Ringold formation.

2. References

Legendre, P., and Legendre, L. (1998). *Numerical Ecology.* Amsterdam, The Netherlands: Elsevier Science.

Stegen, J.C., Lin, X., Fredrickson, J.K., Chen, X., Kennedy, D.W., Murray, C.J., Rockhold, M.L., and Konopka, A. (2013). Quantifying community assembly processes and identifying features that impose them. *ISME Journal* 7**,** 2069-2079.
